# Effect of tracheal tube cuff inflation with alkalinized lidocaine versus air on hemodynamic responses during extubation and post-operative airway morbidities in children: prospective observational cohort study, Ethiopia

**DOI:** 10.1186/s12871-022-01868-2

**Published:** 2022-11-04

**Authors:** Biniam Assefa, Hirbo Samuel, Fissiha Fentie, Tenbite Daniel, Assefa Hika, Bacha Aberra, Belete Alemu

**Affiliations:** 1 Department of Anesthesia, College of Medicine and Health Sciences, Addis Ababa University, Addis Ababa, Ethiopia; 2 Department of Anesthesia, College of Health Science, Aksum University, Aksum, Ethiopia; 3 Department of Anesthesia, College of Health Science, Hawassa University, Hawassa, Ethiopia; 4 Present Address: University Hospital, Hiwot Fana Specialized University Hospital, Harar, Ethiopia

**Keywords:** Air inflations, Alkalinized Lidocaine, Cuff pressure, Endotracheal tube, Laryngotracheal morbidities, Tracheal mucosa

## Abstract

**Background:**

Endotracheal tube with an inflated cuff was used to manage and maintain the airway during general anesthesia in children. When the lateral pressure exerted by an inflated Endotracheal tube cuff on tracheal mucosa exceeds capillary perfusion pressure, patients may complain of cough, sore throat, and hoarseness in the postoperative period. This study aimed to assess the effect of a tracheal tube cuff filled with alkalinized lidocaine versus air on hemodynamic parameter changes during extubation and post-operative airway morbidity in children.

**Methods:**

Institutional based observational prospective cohort study was conducted among 56 elective children; aged 3–13 years, who underwent operation under general anesthesia with cuffed endotracheal intubation for greater than one hour by grouping into the air (group1) and alkalinized Lidocaine (group2) at Tikur Anbessa specialized Hospital. Hemodynamic parameters (Heart rate and Blood pressure) and other variables were measured starting from 5 min before extubation to 24th hours after extubation of the endotracheal tube. A Comparison of numerical variables between study group was done with an independent t-test. Data were expressed in terms of mean ± standard deviation. Categorical data were assessed by Chi-square tests.

**Results:**

Postoperative Sore throat was lower in alkalinized lidocaine group compared to the air group. The mean heart rate at five minutes after extubation was significantly lower in alkalinized lidocaine group (107.29 ± 6.457 beat per minute (bpm)) compared to the air group (122.04 ± 8.809 bpm), with P ≤ 0.001. Systolic blood pressure was also significantly lower in alkalinized lidocaine group (99.64 ± 8.434 millimeters of mercury (mmHg)) compared to the air group (108.21 ± 11.902 mmHg), p = 0.016 at five minutes after extubation.

**Conclusion:**

Alkalinized lidocaine inflated tracheal tubes have shown improved hemodynamic and laryngotracheal morbidities in children.

## Background

Endotracheal tube (ETT) may have a cuff that is inflatable to seal to the trachea and is named cuffed ETT. It may prepare without a cuff and named uncuffed ETT. Uncuffed ETT was preferred over cuffed tubes in children because of concerns about tracheal mucosal damage from excess cuff pressure. However, recent evidence does not support this, and the incidence of post-extubation airway morbidity is not increased when cuffed tubes are used [1].

A cuffed tube allows for measuring tidal volume and proper pulmonary function tests. It also helps to measure accurate end-tidal carbon dioxide and inhalational anesthetic agents. In addition to this proposition, it also serves to decrease the consumption of medical gases, prevent aspiration, reduce pollution of the operating room environment, and maintains the tube midline in the trachea [2–5].

Many anesthesiologists, anesthesia professionals, doctors, and nurses that work in the operation room and ICU checks the cuff pressure by simple palpation of the pilot balloon of the endotracheal tube which is an indirect estimation of pressure in the cuffs. This method of confirmation is not a correct and accurate measurement of the pressure in the cuff [6–10]. When the cuff pressure exceeds the tracheal mucosa capillary perfusion pressure, it can result in tracheal morbidity, loss of mucosal cilia, ulceration, hemorrhage, and tracheal stenosis. Patients may complain of sore throat, hoarseness, and dysphagia in the postoperative period [11–13].

Among many methods that are used to secure the airway, an endotracheal tube is associated with 30–70% of complications during the postoperative period. Coughing, sore throat, dysphonia, dysphagia, dyspnea, and various hemodynamic changes are one of the most expressed complications after endotracheal extubation. Post-operative sore throat contains 50% of the incidents complained about by the vast majority of surgical patients [14, 15].

The incidence of coughing at emergence from general anesthesia reaches in some cases 96% [16]. Sore throat, dysphagia, and dysphonia are frequent and occur in 50% of patients [16, 17]. They are generally badly experienced by the patients. Coughing at emergence from anesthesia reflects intolerance to the endotracheal tube. It may cause many clinical side effects including tachycardia, hypertension, intracranial hypertension, increased intraocular pressure, and surgical complications [16, 18].

Various medications have been shown to reduce coughing, sore throat, dysphonia, dysphagia, and various hemodynamic changes that occur during endotracheal tube extubation and post-extubation; They include lidocaine (Intravenous, Intracuff, Topical, and Tracheal routes), dexmedetomidine, fentanyl, and remifentanil [19–24]. Among these medications, lidocaine is widely available in our set up and some anesthesia professionals use intracuff inflated alkalinized lidocaine for attenuation of pressor response during extubation. Lidocaine is one of the most commonly used drugs for preventing post-operative sore throat, and its efficacy was evaluated in a Cochrane review in 2009 [25]. Nevertheless, the clinical application of the results of this review may still be equivocal, because the route of lidocaine administration was not adequately confined, and its effectiveness on other relevant morbidities was not fully considered.

Hence, the outcome of this study is to compare hemodynamic parameters and laryngotracheal morbidity during extubation and post-extubation among alkalinized lidocaine inflated group and the air inflated group.

## Methods

### Study area

This study was conducted in Tikur Anbessa specialized Hospital (TASH) which is one of the largest teaching and referral hospitals in Addis Ababa, the capital of Ethiopia.

### Study design and period

A prospective institutional-based observational cohort study was employed from October/2019-February/2020.

### Source populatio

All children operated on under general anesthesia with cuff-inflated endotracheal tube intubation at Tikur Anbessa Specialized Referral hospital.

### Study population

All elective children aged 3–13 years who undergo operation under general anesthesia with endotracheal tube intubation for greater than one-hour during the study period.

### Study variables

#### Independent variable

Socio-demographic variables like (Age and sex, Duration of surgery, duration of anesthesia, ASA status, and nature of surgery).

#### Dependent variable

Hemodynamic response changes **(**HR, SBP, AND DBP), cough, sore throat, and hoarseness.

### Inclusion criteria

All Children aged 3– 13 years, of ASA physical status 1 or 2 undergoing elective surgery under general anesthesia requiring oral tracheal intubation were considered eligible for inclusion in the study.

### Exclusive criteria

Those with oropharyngeal or neck malformations, patients who had previously required tracheal intubation or a tracheostomy, the presence of stridor or dysphonia, and those requiring surgery to the neck, larynx, or oropharynx.

### Sample size and sampling technique

The sample size was calculated with the incidence of sore throat that was observed in the post-anesthesia care unit after tracheal extubation, by using the study done in Brazil [14]. Taking these, the sample size was calculated based on double population proportion formula. For a type, one error of 0.05 and type two error of 0.2 with a power equal to 80% and confidence interval of 95%. thus, the sample size was 56 patients with 28 patients in each group.

P_1_(1−p_1_)+p_2_(1−p_2_)

(P1−p2)^2^

0.02(1−0.02) + 0.2(1−0.2) *2.71 = 24.98

(0.2−0.02)^2^

n = Sample size in each group

α = significance level (1.96)

1-β = power of study at 80% (0.84)

q1= 1−p1

q2=1−p2

p1 = incidence of postoperative sore throat in the lidocaine group.

p2 = incidence of postoperative sore throat in the air group.

It was approximated to 28 for each group, when 10% of contingency is included by assuming a loss to follow up, a total sample of 56 patients or 28 patients per group was required.

### Procedure

From situational analysis, during the study period, 165 children were estimated to undergo surgery under general anesthesia with cuffed endotracheal tube intubation in the hospital. With systematic random sampling, every 3rd (165/56 = 2.9) elective patient from the schedule, who fulfilled inclusion criteria, and volunteers were recruited to take part in the study. The first sample was selected by lottery method from scheduled patients listed on the display board on the first day of the study period, the patients were not randomized for anesthetic management.

All patients were visited in the morning before surgery; informed consent was obtained, and follow-up should be started. On arrival in the operating room, all patients were attached to ECG, pulse oximetry, temperatures, and non-invasive blood pressure monitoring. At the induction of anesthesia, a patient breathes 100% oxygen via a facemask, and then, all patients were anesthetized according to a standard protocol and preferences of the respective anesthetists, who handle the case.

All patients were pre-oxygenated and received thiopentone for induction and suxamethonium, depolarizing muscle relaxant for facilitating laryngoscopy. Laryngoscopy was performed and the trachea was intubated with a standard cuffed endotracheal tube according to the patient’s age. The endotracheal tube cuff inflation whether alkalinized lidocaine or air was, under the preferences of anesthetists who manage (handle) the case. The cuff was inflated until there was no air leak around the tube when positive pressure was applied. Alkalinized lidocaine used to inflate the tubes was prepared by mixing 19 ml of lidocaine 1% and 1ml of sodium bicarbonate solution. The total amount of alkalinized lidocaine used for inflation was 1ml to 2ml.

Isoflurane was the maintenance agent that was used in both groups, Vecuronium was the muscle relaxant that was used for anesthetic maintenance in all patients, and the reversal agent was neostigmine.

### Data collection

The evaluated demographic and clinical characteristics of the study participants were: Gender, age, ASA physical status, nature of the surgery, duration of surgery, and duration of anesthesia. Evaluated hemodynamic parameters were systolic blood pressure, Heart rate, and Diastolic Blood Pressure. The assessed Postoperative laryngotracheal morbidities were: Postoperative cough, hoarseness, and sore throat.

### Data collection technique and instrument

Data were collected using a pretested observational checklist. Data collectors were one bachelor’s degree holder anesthetist and two bachelor’s degree holders nurse and they were supervised by one master’s degree holder anesthetist. Questionnaires were prepared in both English and Amharic languages and it was divided into three parts, the first one was filled in the preoperative and intra-operative time and collected by one trained BSc anesthetist and the second one was PACU record going to be recorded by a PACU nurse and the third one was filled in the ward which was filled by trained ward nurse [26].

### Data processing and analysis

The Data was entered using the Epi-Info version 7.0 and clean-up has been made to check accuracy, consistency, and errors. Errors identified were corrected and finally, the data was transported to SPSS Version 20 for analysis. The normality of the data distribution was analyzed by the Shapiro-Wilk test for non-normally distributed data and the histogram with bell-shape for normally distributed data. while homogeneity of variance was assessed using Levene’s test for equality of variance. Numeric data were described in terms of mean ± SD for symmetric data [26].

**Operational Definition:**

#### Sore throat

A constant pain or discomfort in the throat independent of swallowing.

#### Hoarseness

Difficulty in speaking or pain in speaking.

#### Cough

A sudden reflex that forces air out of the throat.

#### Alkalinized Lidocaine

Is a solution that is prepared by mixing 19 ml lidocaine 1% or 2% with 1 ml sodium bicarbonate 8.4% solution.

#### ASA status

a surgical risk stratification validated by the American Society of Anesthesiologists.

## Results

### Socio-demographic status

A total of 56 patients participated in this study 28 in the air (group 1) and 28 in alkalinized lidocaine (group 2) filled cuffed endotracheal tube respectively and all of the participants were included in the study.

The demographic status and clinical characteristics of the data were comparable between groups with a p-value greater than 0.05. (Table 1)

**Table 1:** Demographic and clinical characteristics of the study participants

		Air group n (%)	Lidocaine group n (%)	P-value
Gender	Male	13 (44.8%)	16 (55.2%)	0.422*^a^
	Female	15 (55.6%)	12 (44.4%)	
Nature of surgery	General surgery	14 (40.00%)	21 (60.00%)	0.097*^a^
	Urologic surgery	14 (66.7%)	7 (33.3%)	
ASA status	ASA 1	21 (56.8%)	16 (43.2%)	0.158*^a^
	ASA2	7 (36.8%)	12 (63.2%)	
Age (in years)	In mean and SD	7 ± 3 years	8 ± 3 years	0.177**^b^
Duration of surgery (in minutes)	In mean and SD	111 ± 28 min	122 ± 20 min	0.098**^b^
Duration of anesthesia (in minutes)	In mean and SD	129 ± 29 min	140 ± 22 min	0.113**^b^

*^a^: P - value obtained from chi-square test,

**^b^: P – value obtained from independent t-test

SD = Standard Deviation, n = frequency, % =proportion

### Mean heart rate between the groups

The results of the independent t-test show that the mean heart rate (Bpm) at 5 min before extubation has significantly lower in the lidocaine group compared to the air group. Similarly, the results of mean heart rate (Bpm) at 5 min after extubation also show a significant decrease in the lidocaine group compared to the air group. (Table 2)

**Table 2:** Mean heart rate of the study participants

Heart rate (Bpm)	Air group (Mean ± SD)	Lidocaine group (Mean ± SD)	*P-value
5 min before extubation	115 ± 7	110 ± 9	0.036*^a^
5 min after extubation	122 ± 9	107 ± 6	< 0.001*^a^

*^a^ P-value was obtained from an independent t-test

### Mean systolic blood pressure between the groups

The results of the independent sample t-test of this study show that the mean systolic blood pressure(mmHg) at 5 min before extubation has significantly lowered in the lidocaine group compared to the air group. Similarly, the results of mean systolic blood pressure (mmHg) at 5 min after extubation in this study show a significant decrease in the lidocaine group compared to the air group. (Table 3)

**Table 3:** Mean SBP of the study participants

Systolic blood pressure (mmHg)	Air group (Mean ± SD)	Lidocaine group (Mean ± SD)	P- value
5 min before extubation	109 ± 9	103 ± 7	0.021*^a^
5 min after extubation	108 ± 12	100 ± 8	0.003*^a^

*^a^: P-value was obtained from an independent t-test

### Mean diastolic blood pressure between the groups

The results of the independent t-test show the mean diastolic blood pressure (mmHg) at 5 min before extubation has decreased in the lidocaine group compared to the air group. (Table 4)

**Table 4:** Mean DBP of the study participants

Diastolic blood pressure (mmHg)	Air group (Mean ± SD)	Lidocaine group (Mean ± SD)	* P- value
5 min before extubation	72 ± 10	66 ± 11	0.04*^a^
5 min after extubation	73 ± 9	66 ± 9	0.003*^a^

*^a^: P-value was obtained from an independent t-test

Similarly, the mean diastolic blood pressure(mmHg) at 5 min after extubation show a significant decrease in the lidocaine group compared to the air group. (Table 4)

### Post-operative laryngotracheal morbidities

The incidence of postoperative coughs recorded in the PACU show significant increments with the proportion of 70% occurrence in the air group compared to 30% in the lidocaine group. The p-value of difference between the group show 0.026, which is significant.

The incidences of post-operative Hoarseness at extubation in the PACU were lower in the lidocaine group with a proportion of 44.4% compared to 55.6% in the air groups. The p-value between the group has not shown a significant difference, which is 0.567. Meanwhile, the incidence of postoperative hoarseness at 8 h shows significantly lowered with the proportion of 30.4% in the lidocaine group compared to 69.6% in the air group. The p-value between the group shows 0.014, which is significant.

**Fig. 1:**
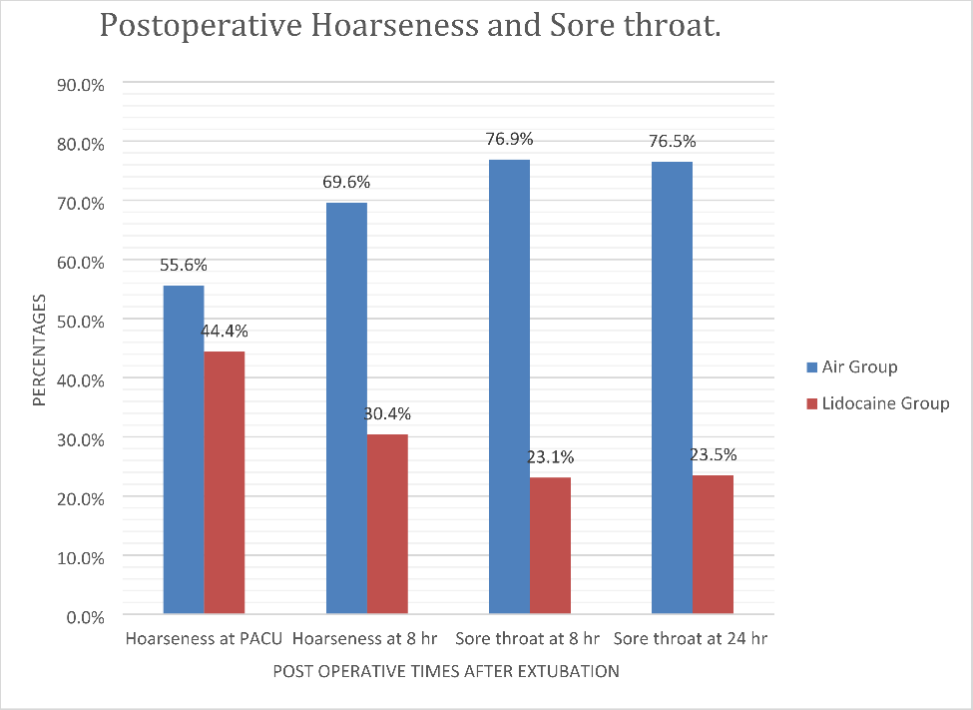
Proportion of postoperative hoarseness and sore throat within the air and alkalinized lidocaine group of the study participants

The incidence of postoperative sore throat at 8 h has significantly decreased in alkalinized lidocaine group with the proportion of 23.1% compared to the air group which shows 76.9% and a p-value of 0.027, which is significant. Similarly, the results of chi-square show the incidence of postoperative sore throat that was measured at the 24 h has lowered in the lidocaine group with a proportion of 23.5% compared to 76.5% in the air group with a p-value of 0.009, which is a significant (Fig. 1).

## Discussion

Administration of general anesthesia using cuffed endotracheal tubes is a common practice in our setup for the management of general anesthesia during major surgeries. Among many complications that occur during extubation and the post-operative period; cough, sore throat, and hoarseness are highly associated with endotracheal tube cuffs. These complications are due to direct trauma or damage to the structures of the tracheal mucosa and throat. Post-operative cough at the time of emergence from general anesthesia and recovery room can lead to serious complications like hypertension, cardiac arrhythmias, myocardial ischemia, surgical bleeding, bronchospasm, and raised intra cranial and ocular pressures [27].

Even though, there are studies that have compared the effect of a tracheal tube cuff filled with alkalinized lidocaine versus air on laryngotracheal morbidity in children. In our country still, we don’t have such a tracheal tube cuff pressure measurement instrument and still, there is a discrepancy in using adjuvants for controlling this problem.

Our results show a significantly lower heart rate at 5 min before extubation in the lidocaine group compared to the air group, with p-values of 0.036. This result is comparable with the heart rate difference seen in the study done by Soares et al. in children, showing a p-value of < 0.001 among the group [28].

Our finding shows the heart rate at 5-minute after extubation significantly decreased in the lidocaine group compared to the air group, with a p-value of < 0.001. This result is comparable with the study done by Soares et al. that shows a reduction in the mean heart rate after extubation in the lidocaine group compared to the air group, with a p-value of 0.007 among the groups [28]. Additionally, the study done by using lidocaine and air in the adults’ population which measured heart rates at 1, 2, 5, 10, and 30 min after extubation show a similar result to our study with the mean heart rate being lower in the lidocaine group at 5 min after extubation with a p-value of 0.003 [29].

In contrast to our results, the study done by Benzadi et al. that measured the heart rate from 1 to 5 min after extubation shows no significant difference among the group with a p-value of 0.942 [30]. This is due to their study using shorter-duration surgical procedures.

Our result shows systolic blood pressure at 5 min before extubation was significantly lowered in the lidocaine group compared to the air group, with a p-value of 0.021. This result is comparable with the study done by Soares et al. that shows a significant decrease in mean systolic blood pressure in the lidocaine group when compared to the air group; with a p-value of < 0.021 [28]. This effect is due to lidocaine-inflated cuffs being more tolerable than air-filled endotracheal tubes.

Our study shows the systolic blood pressure at 5 min after extubation shows a significant decrease in the lidocaine group compared to the air group, with a p-value of 0.003. This result is comparable with the study done in children by Soares et al. which shows a significant decrease in the mean of SBP in the lidocaine group (110.9 ± 15.7) compared to the air group (108.7 ± 17.1) with a p-value of 0.022 between the groups [28]. This is due to lidocaine-containing cuffs being more tolerable than air-filled cuffs.

Our study shows diastolic blood pressure at 5 min before extubation significantly decreased in the lidocaine group compared to the air group, with a P-value of 0.04. Similarly, the diastolic blood pressure at 5 min after extubation was also decreased in the lidocaine group compared to the air group, with a p-value of 0.003 between the groups. This result is comparable to the study done by Soares et al. on children shows diastolic blood pressure among the groups was significantly different, with a p-value of 0.019 [28].

Cough is a protective mechanism induced by rapidly adapting stretch receptors in the tracheal mucosa; are believed to be irritant receptors involved in the cough reflex [31].

Our result shows the incidence of postoperative cough that was recorded after extubation in the PACU was significantly higher in the air group compared to the lidocaine group; with a p-value of 0.026. This result is comparable with the study done by Ahmed et al. in children which founds incidence of postoperative cough in PACU was higher in the air group compared to lidocaine; with a p-value of 0.048 among the groups. Contrary to our result Soares et al. show that the incidence of postoperative cough in PACU has no significant difference between the group; with a p-value of 0.419. These may be due to the use of large amounts of opioids they use at the end of the operation, and the strict extubation airway manipulation technique they used during their study [28].

Our result shows the incidence of postoperative hoarseness in the PACU has a lower proportion of occurrence in the lidocaine group compared to the air group; with a p-value of 0.567.

This result is in line with a study done on children by Soares et al. and Ahmed et al. show no significant difference in the incidence of hoarseness at PACU between the groups with a p-value of 0.308 and 0.667 respectively [28, 32]. These may be due to hoarseness caused by mechanical trauma that occurred during endotracheal intubation; the symptom is noticed most of the time after 8 h of post-extubation.

Our result shows the incidence of postoperative hoarseness at 8 h after extubation has significantly higher in the air group compared to the lidocaine group; with a p-value of 0.014. This result is comparable with Benzadi et al. founds p-value of difference between the groups was < 0.001 [30].

Contrary to our study Soares et al. and Ahmed et al. show that postoperative hoarseness in children at 8 h has no difference between the groups with a p-value of 0.448 and 0.298 respectively. This may be due to the cuff pressure monitored throughout the operation being constant and the strict extubation airway manipulation technique they used during their study [28, 32]. The activation of pain receptors in the throat mucosa due to the compression of the endotracheal tube cuff from overinflation and the tube itself can cause postoperative sore throat. Our finding shows the incidence of postoperative sore throat observed at 8 h after extubation was higher in air groups compared to the lidocaine group, with a p-value of 0.027. Our study is comparable with Soares et al. who stated in their study the proportion of sore throat at 8 h after extubation was 72% in the air group and 22% in the lidocaine group with a p-value of 0.014 [28].

Our result shows the incidence of postoperative sore throat 24 h after extubation was higher in the air group compared to the lidocaine group. The p-value of the difference between the groups was 0.009. Our study is in line with Ahmed et al. showed that the postoperative sore throat at 24 h significantly decreased in the lidocaine group with a proportion of (4%) compared to the air group (28%); with a p-value of 0.048 [32]. Many other studies in adults and children show sore throat decreased significantly in lidocaine-inflated tubes [28].

Regarding the lidocaine group, the decreased postoperative sore throat is due to continuous application of local anesthetic to the tracheal mucosa may reduce the occurrence of post-extubation laryngo tracheal morbidity. In vitro study showed that a local anesthetic-filled cuff diffused through the cough membrane in a dose-dependent fashion [33]. Relatively low concentrations of lidocaine could block different sensory tracheal receptors and suppress their action potentials. The reduction in the sore throat as a whole is related to the anti-inflammatory action of lidocaine. The half-lives of topical lidocaine in children aged 3 months to 9.5 years are 109 min [33].

### Strength of the study

The study group was homogenous (children aged 3–13 years), undergoing elective surgery under general anesthesia with a cuffed endotracheal tube. Good, follow-up of study participant for 24 h post-extubation. It gives clues for further research in a resource-limited setting like our country (Ethiopia).

### Limitation of the study

Failure to measure the cuff pressure of the endotracheal tube cuff inflation was a major limitation of our study. Unable to randomize and double-blind, since it is an observational study was also another limitation.

## Conclusion

Our study analysis and data show endotracheal tubes that were inflated with alkalinized lidocaine instead of room air reduce the postoperative complication associated with endotracheal tube cuff; fewer incidences of postoperative sore throat and hoarseness. In addition, it can promote reduced hemodynamic changes during extubation. We recommend anesthetists to use alkalinized lidocaine into endotracheal tube cuffs, in children undergoing general anesthesia with cuffed endotracheal tube intubation for elective procedures.

## Data Availability

The datasets generated and/or analyzed during the current study are available to the corresponding author and will be submitted upon request for securing confidentiality.

## List of abbreviations

AAU: Addis Ababa University
ASA: American Society of Anesthesiologists
Bpm: beat per minute
CP: Cuff Pressure
DSB: Diastolic Blood Pressure
ECG: Electrocardiograph
ETGA: Endotracheal tube intubation in general anesthesia
ETT: Endotracheal tube
GA: General Anesthesia
HR: Heart Rate
ID: Internal diameter
PACU: Post Anesthesia Care Unit
POST: Postoperative Sore Throat
SBP: Systolic Blood Pressure
TASH: Tikur Anbessa Specialized Hospital
